# Postpartum Necrotizing Myometritis

**DOI:** 10.1002/ccr3.72339

**Published:** 2026-03-19

**Authors:** Jiangyan Xie

**Affiliations:** ^1^ Department of Obstetrics Hainan Women and Children Medical Center Haikou China

**Keywords:** case report, necrotizing myometritis, postpartum hemorrhage, uterine arterial embolization, uterine compression suture

## Abstract

We present a case of postpartum necrotizing myometritis, a rare complication that often occurs following transcatheter arterial embolization for the treatment of postpartum hemorrhage, and its etiology is closely associated with uterine ischemia and bacterial infection. Due to the nonspecific clinical manifestations, its diagnosis is often challenging. The essential treatments are maintaining uterine cavity patent, debriding the infected and necrotic tissues, and providing aggressive anti‐infective therapy. Hysterectomy could be performed if necessary. Failure to control the disease timely may be life‐threatening.

## Key Clinical Message

Postpartum necrotizing myometritis is a rare but life‐threatening complication following conservative surgical interventions for postpartum hemorrhage. High clinical suspicion, early contrast‐enhanced MRI, and prompt multidisciplinary management are crucial. This case underscores the need to balance effective hemostasis with uterine perfusion preservation and aggressive infection control to avoid hysterectomy and reduce mortality.

## 1 Introduction

Postpartum necrotizing myometritis is a severe disease with a poor prognosis that has only been reported in a few cases. It often occurs after transcatheter arterial embolization (TAE) for postpartum hemorrhage (PPH), and there are also reports after uterine compression suturing and pelvic vessel ligation [1]. Postpartum necrotizing myometritis mainly presents nonspecific symptoms such as abdominal pain, fever, and abnormal vaginal bleeding, which often make early diagnosis challenging. Knowing risk factors, clinical features, and treatment methods of necrotizing myometritis can help obstetricians recognize the condition earlier and control it effectively.

## 2 Case History/Examination

A 39‐year‐old female, gravida 1, para 0, with a pregnancy at 38 weeks and 1 day, delivered a male infant naturally, weighing 3300 g. Due to excessive vaginal bleeding caused by partial placental separation after delivery, manual placental extraction was performed. The placenta was sent for pathological examination. The patient had severe PPH due to uterine atony and was treated with oxytocin, carboprost tromethamine, ergometrine maleate, as well as the placement of an intrauterine balloon catheter. However, as the vagina continued to bleed slightly, an emergency exploratory laparotomy was performed under general anesthesia. During the surgery, the uterus was pale, contracted poorly, and appeared sac‐like. A B‐Lynch suture, bilateral uterine artery ligation, and intrauterine balloon tamponade were performed. The total blood loss was 5350 mL after delivery, with transfusion of 20.5 U of red blood cell suspension, 3000 mL of fresh frozen plasma, 32 U of cryoprecipitate, 6 g of fibrinogen, and 1 platelet treatment. The patient was transferred to the Intensive Care Unit (ICU) for treatment after the operation. Adrenaline was administered to maintain blood pressure.

## 3 Differential Diagnosis, Investigations, and Treatment

In the early postoperative period, the antibiotic regimen was changed from cefazolin sodium to intravenous cefoperazone sodium and sulbactam sodium combined with metronidazole for infection prevention. However, routine and biochemical analysis of the postoperative abdominal drainage fluid suggested the presence of infected ascites, leading to a further adjustment of the anti‐infection regimen: cefoperazone sodium and sulbactam sodium were discontinued and replaced with moxifloxacin hydrochloride injection. In addition, the placental pathology results revealed focal mild acute deciduitis and early villitis.

On postoperative day 5, the patient's temperature rose to 38.5°C, accompanied by significant uterine tenderness, clinically suggesting possible uterine infection. As her blood pressure still required vasopressor support, a diagnosis of septic shock was considered. Metronidazole was discontinued and vancomycin was added to intensify anti‐infective therapy. Ultrasound examination revealed a 33 × 31 mm anechoic area in the uterine cavity, suggesting possible pus accumulation. Ultrasound‐guided curettage was planned but abandoned due to cervical necrosis preventing probe insertion.

On postoperative Day 7, the patient's temperature remained fluctuating around 38°C. Chest CT revealed atelectasis pneumonia with pleural effusion, and pelvic CT showed multiple high‐density shadows in the uterine wall, suggesting possible hemorrhage. Sputum culture grew *Klebsiella oxytoca* , and piperacillin‐tazobactam was added based on drug susceptibility testing. The patient's temperature initially decreased but subsequently rose again; piperacillin‐tazobactam was discontinued and replaced with imipenem‐cilastatin, after which the temperature increased to 39.8°C.

**FIGURE 1:**
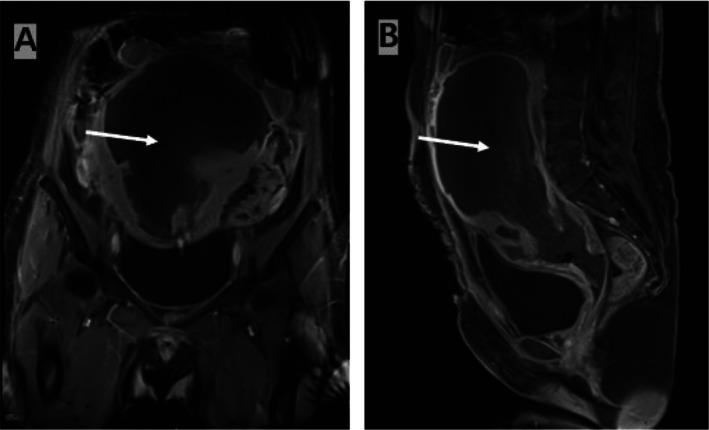
Pelvic enhanced MRI, arrows showing unenhanced necrotic myometrium: (A) coronal position; (B) sagittal position.

On postoperative Day 8, contrast‐enhanced pelvic MRI revealed significant thickening and edema of the uterine wall with heterogeneous enhancement (hypoenhancement), suggesting possible uterine ischemic necrosis. To confirm the diagnosis, ultrasound‐guided myometrial biopsy was performed, obtaining a 2 × 0.5 × 0.5 cm tissue specimen for pathological examination. The following day, repeat contrast‐enhanced pelvic MRI indicated diffuse swelling of the uterus and cervix with necrotic changes, involving approximately 70%–80% necrosis (Figure 1). Pathological examination of the biopsy specimen showed: most of the submitted tissue exhibited degeneration and necrosis with hemorrhage, with abundant neutrophil‐predominant inflammatory cell infiltration; smooth muscle‐like contour shadows were visible within the degenerated necrotic tissue, and a small amount of fibrous tissue and smooth muscle‐like tissue was seen at the periphery, confirming the diagnosis of necrotizing myometritis.

## 4 Conclusion and Results (Outcome and Follow‐Up)

**FIGURE 2:**
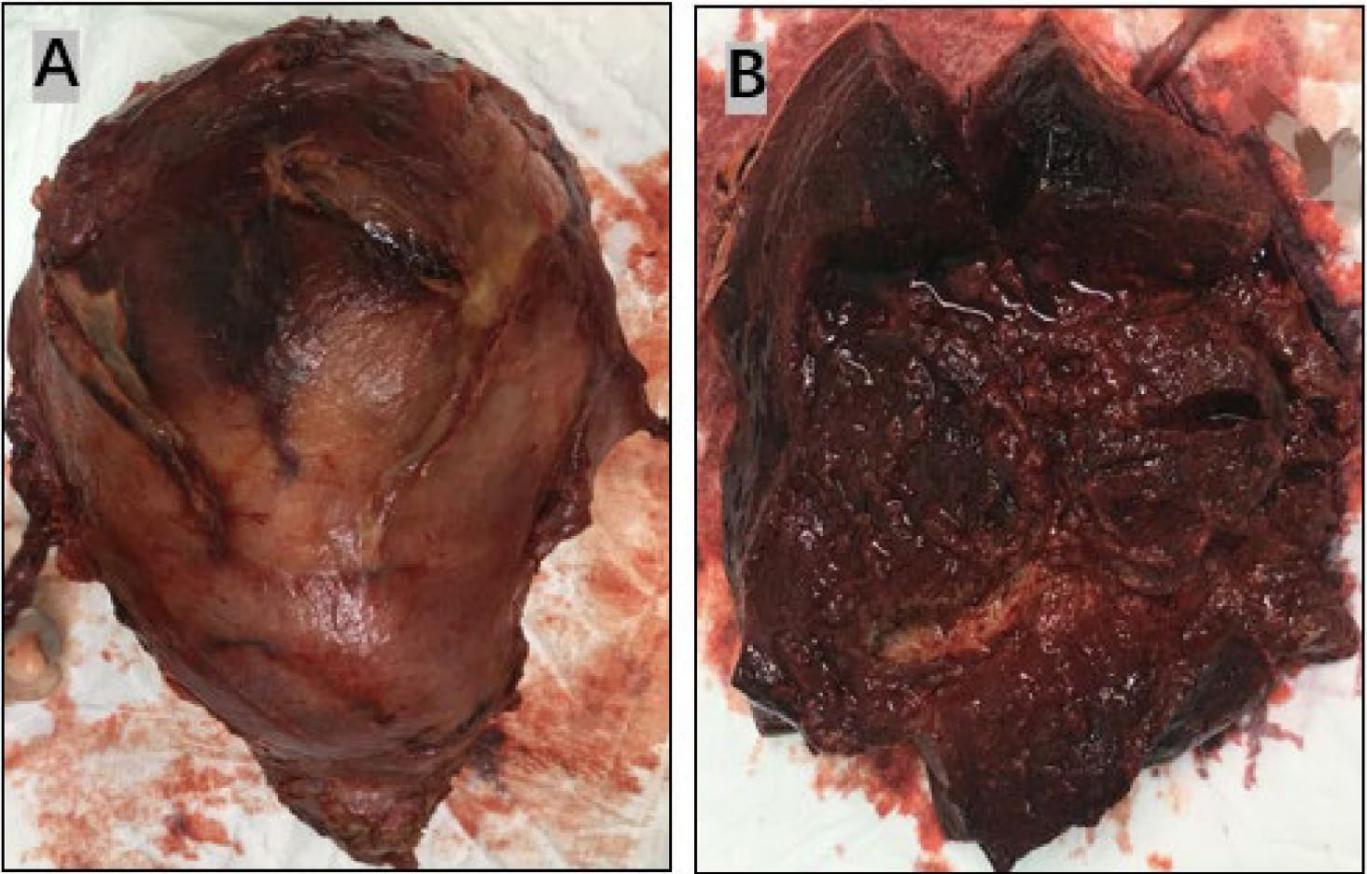
The uterus removed by surgery: (A) the uterus is dark red; (B) the incised uterus: Myometrium and endometrial tissue were dark red and rotten.

**FIGURE 3:**
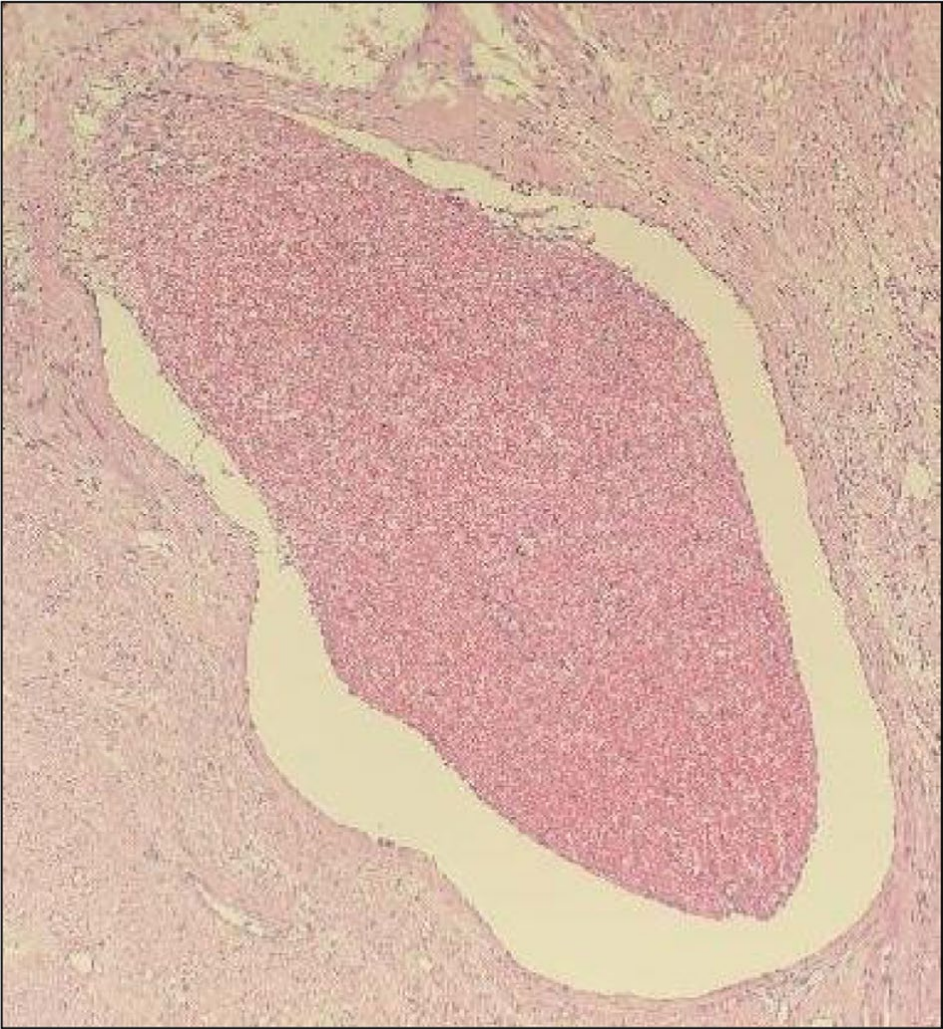
Histopathological examination of the myometrium (H&E stain, ×100) showing a dilated and congested small blood vessel filled with numerous red blood cells. Surrounding myometrial fibers show extensive degeneration and necrosis, with focal and diffuse infiltration of a large number of neutrophils, accompanied by interstitial congestion and edema.

After multidisciplinary consultation, on postoperative Day 19, a second laparotomy was performed, with total hysterectomy and bilateral salpingectomy. Intraoperatively, the uterus was about 18 × 16 × 10 cm, soft and dark red. On sectioning, the uterine muscle was found to be brittle (Figure 2). Postoperative pathology (Figure 3) showed most of the endometrium and myometrium had undergone bleeding, degeneration and necrosis, with inflammatory reactions, dilated blood vessels, thrombi and intravascular degenerated and necrotic tissue. The patient's body temperature returned to normal on the third day after the second operation, and she was discharged on the 10th day.

## 5 Discussion

Postpartum necrotizing myometritis is a rare clinical disease closely related to uterine ischemia and bacterial infection, with generally poor prognosis and potential death risk. TAE, uterine compression sutures, and pelvic vessel ligation are important conservative surgeries for PPH with reliable hemostatic effects [2], but they can also lead to uterine ischemia and necrosis. The occurrence of TAE‐induced uterine necrosis is mainly related to the formation of collateral circulation, the embolization technique, and the size of embolization particles. Some scholars [3, 4] suggest using absorbable particles with a diameter of 500–900 μm during TAE to reduce distal uterine ischemia. In terms of embolization technique, free‐flow targeted embolization and superselective uterine artery embolization are recommended to prevent unnecessary vessel occlusion. Uterine compression sutures achieve hemostasis through direct mechanical compression of the uterine sinusoids, with a total effectiveness of 97% [5], including B‐Lynch sutures, Hayman suture method, Cho square suture [6], double B‐Lynch sutures [7], and modified B‐Lynch sutures [1]. The tension of the uterine compression sutures is both the key to successful hemostasis and closely related to the occurrence of complications [8]. If the sutures are too tight, too dense, or placed incorrectly, they can block blood flow to the uterine muscle layer, leading to intrauterine hematoma, pyometra, adhesions, and uterine necrosis [1]. In this case, multiple hemostatic techniques were employed, such as the uterine B‐Lynch suture and bilateral uterine artery ligation, along with the placement of an intrauterine balloon. While these measures controlled PPH, they also increased the risk of infection and uterine ischemic necrosis. The possibility of a technical failure of the hemostatic sutures or ligatures was considered but ruled out based on the intraoperative findings of a diffusely pale and poorly contracted uterus, along with imaging evidence of widespread necrotic changes rather than focal hematoma or active bleeding. This supports that the necrosis was primarily due to the combined effects of ischemia and infection. To reduce complications from uterine compression suturing, some studies have suggested using monofilament absorbable sutures that cause less damage to the uterine wall [9], avoiding excessive and dense suturing during surgery [10], and removing sutures laparoscopically or using removable sutures [8, 11]. These approaches are worth considering in clinical practice.

Infection is crucial for the development of necrotizing myometritis. Recent literature emphasizes the interplay between uterine ischemia and ascending infection in the pathogenesis of necrotizing myometritis [1]. Infection pathogens can induce the aggregation of platelets and white blood cells, leading to endothelial cell damage and capillary blockage, which in turn further reduces blood supply and accelerates tissue necrosis [12]. Additionally, in ischemic tissues, antibiotics are unable to achieve effective blood concentrations locally, which can lead to bacterial proliferation and worsen the infection. In this case, multiple vaginal examinations were performed during the trial of labor, and after delivery, manual placental removal and intrauterine balloon tamponade were carried out, increasing the risk of ascending vaginal infection. Subsequently, due to intractable PPH, uterine compression suturing and uterine artery ligation were performed, which compromised uterine blood flow and exacerbated intrauterine infection. All of these factors contributed to the development of necrotizing myometritis.

The diagnosis of necrotizing myometritis is usually based on clinical symptoms and imaging examinations. Typical symptoms include lower abdominal pain, fever, vaginal bleeding, and abnormal discharge [13]. Imaging features involve uneven uterine muscle layer echoes, peripheral enhancement, and gas echoes in necrotic areas [14]. When uterine necrosis is suspected, ultrasound is the first‐line imaging tool, yet it has a high miss‐diagnosis rate and often needs CT or MRI confirmation [15]. In this case, the patient had recurrent fever, fundal tenderness, and unstable blood pressure. Clinical symptoms aligned with necrotizing myometritis. But initial ultrasound and noncontrast CT showed no uterine necrosis. It was not until contrast‐enhanced MRI that possible uterine ischemic necrosis was indicated. Later, pathological tests confirmed necrotizing myometritis.

Owing to the limited number of necrotizing myometritis cases, no standardized treatment protocol is available at present [16]. The key to treatment is to ensure patency of the uterine cavity, remove infected and necrotic tissue, and actively treat infections, with hysterectomy performed when necessary [14]. The surgery generally involves total hysterectomy [17], although there are also reports of only removing necrotic tissue [14] or employing fertility‐sparing techniques in early‐presenting cases [18]. In this case, the patient had symptoms such as abdominal pain and fever and underwent active antibiotic treatment. The antibiotics were later changed based on the sputum culture and sensitivity results, but the treatment was ineffective due to compromised uterine blood supply and poor uterine cavity drainage, leading to a total hysterectomy.

Postpartum necrotizing myometritis is rare and has a poor prognosis, severely threatens women's fertility, and carries a potential risk of death. For patients with risk factors, early warning and proactive condition control are vital. Due to physiological and pathological changes during pregnancy and the puerperal period, clinical manifestations are often nonspecific. Various indicators and lab test results differ from nonpregnant periods, making early diagnosis difficult and prone to missed diagnoses. Therefore, a multidisciplinary team should be organized for joint management to prevent disease delay.

## Author Contributions

**Jiangyan Xie:** conceptualization, data curation, funding acquisition, investigation, methodology, project administration, visualization, writing – original draft, writing – review and editing.

## Funding

This work was supported by 2024 Hainan Health Science and Technology Innovation Joint Project (Grant WSJK2024MS166), Supported by The Excellent Talent Team of Hainan Province (Grant QRCBT202121).

## Consent

Written informed consent was obtained from the patient to publish this report in accordance with the journal's patient consent policy.

## Conflicts of Interest

The author declares no conflicts of interest.

## Data Availability

The data that support the findings of this study are available on request from the corresponding author. The data are not publicly available due to privacy or ethical restrictions.
